# Avazum app usability testing

**DOI:** 10.1590/2317-1782/20232022103en

**Published:** 2023-10-09

**Authors:** Hionara Nascimento Barboza, Marcillyo Carneiro de Lima, Rubens Jonatha dos Santos Ferreira, Marine Raquel Diniz da Rosa, Ana Loisa de Lima e Silva Araújo, Angélica de Souza Galdino Acioly

**Affiliations:** 1 Departamento de Fonoaudiologia, Universidade Federal da Paraíba - UFPB - João Pessoa (PB), Brasil.; 2 Departamento de Design, Universidade Federal da Paraíba - UFPB - João Pessoa (PB), Brasil.

**Keywords:** Mobile Applications, Tinnitus, Evaluation Study, User-involved Design, Technological Development and Innovation Projects

## Abstract

**Purpose:**

To verify the effectiveness, efficiency, and satisfaction in the usability test of the tinnitus assessment application.

**Methods:**

This is a descriptive usability test study that assessed the satisfaction, effectiveness, and efficiency of the application. The test was carried out virtually via Google Forms. First, the participants received instructions on how to access and use Avazum, through texts and illustrative images. Afterward, the users used the application, observing its functions and usability. Next, the participants answered the usability questionnaire. Statistical analysis was performed using the Statistical Package for the Social Sciences, version 21.0, to perform descriptive analysis such as frequency, percentages of data in the System Usability Questionnaire (SUS), and analysis in the Net Promoter Score (NPS).

**Results:**

Thus, the overall mean SUS score was consistent with satisfactory usability, which implies that the application does not present serious usability problems. Also, the overall NPS percentage indicated very good user satisfaction, with a good percentage of promoting users. As far as effectiveness is concerned, it was found that Avazum reached its goals, besides being efficient, as it uses clear language and is comfortably used.

**Conclusion:**

Avazum obtained very good satisfaction from users, in addition to achieving its goals. It proved to be effective, has clear language, and is comfortably used, adducing efficiency in the multidisciplinary evaluation of tinnitus.

## INTRODUCTION

The scientific revolution has brought to society a wider look at how to deal with the world, providing advances in all areas. Technology is a result of modern science, combining techniques and methods to develop it and make it as powerful and useful as it is today^(1)^. Technology became a great ally in the field of health, with advances in procedures, techniques, healing, innovation in health education, healthcare, and so forth. The quick development and generalized use of mobile technology have been expanding opportunities for health activities, ushering in a new era - that of mobile health (mHealth)^(2)^.

Technological innovations are becoming one of the main allies in all areas of speech-language-hearing therapy, including health processes, products, and services. Currently, various applications and pieces of software are being developed to innovate therapy, assessment, health education, and so on. Concerning tinnitus, various applications are currently available to help its treatment, such as sound therapy and cognitive behavioral therapy. Applications with greater availability on platforms are those focused on therapy, but few of them have functions aimed at multidisciplinary assessments, with differential referral functions. Moreover, most applications did not undergo usability tests^(3)^.

Avazum is an application developed to help in the initial assessment of tinnitus, helping screen and refer users to the necessary professionals, providing detailed assessment according to the need in each case, and hints and instructions on tinnitus care. The application has three main interfaces, namely: the registry screen, the assessment screens, and the screen with results, referrals, hints, and guidance. It was developed by an interdisciplinary team, involving speech-language-hearing therapists, programmers, and designers, members of two research groups on health technology innovation, and a study and research group on tinnitus of the Federal University of Paraiba. The application has undergone all development phases, and its usability test is conducted in this study.

A usability test is a method to verify interface functioning in a digital platform. It is used in websites, applications, and other tools, leading real users to do certain tasks. After its development, its usability and main difficulties are analyzed. Usability tests record the best results for future updates, minimizing the costs of technical support to users, increasing sales, and predicting new products with fewer usability problems.

The usability test is an indispensable requirement to develop technological innovation products, as it analyzes the necessary information to detect occasional usability problems and, consequently, furnishes tools to deliver a quality product to users^(4)^.

Evidence-based health practices are essential to provide excellent services with proven results. When developing innovative products in this area, it is essential to understand the needs of users to meet the desired experience with objectivity and quality in the target functions.

Hence, this study aimed to verify the effectiveness, efficiency, and satisfaction of the usability test of the tinnitus assessment application.

## METHODS

### Avazum characterization

The application initially has a user registry screen with an e-mail and password for their login. After registering, users are directed to interactive assessment screens, with all aspects related to the symptoms - e.g., how it began, type and sound characterization and location - and audio-visual resources. Later, they are directed to sessions on the habits that worsen or improve tinnitus perception, measuring discomfort with a visual analog scale. After finishing all assessment stages, users have access to the results, indicating possible symptoms and associated causes. It also indicates professionals according to each case’s specificities, based on referral descriptors, relating the symptoms with specific professionals in each area, including speech-language-hearing therapists, otorhinolaryngologists, psychologists, physical therapists, and nutritionists. In the end, it provides hints and guidance on tinnitus care related to physical and mental health. The application’s registry number in INPI is BR512020001425-9. Further details on the application will be provided in another study entitled “Developing Avazum: An interactive tinnitus assessment application”, yet to be published.

### Study design and instruments used

This is a descriptive study of the usability test of a tinnitus assessment application (Avazum).

The instruments used in the usability research addressed the application’s satisfaction, efficiency, and effectiveness (Annex A). Satisfaction was assessed with an empirical method, using the System Usability Questionnaire (SUS) and Net Promoter Score (NPS), while its efficiency and effectiveness were assessed with an analytical method, considering heuristic principles for the application.

- Effectiveness

The assessment aimed at collecting information on meeting the users’ objectives and the product’s capacity to do that for which it was designed. Hence, it approaches the assessment, guidance, and referral of tinnitus patients. Efficiency and effectiveness responses are observed based on the presence or absence of problems to be solved. If the user perceives a problem, they indicate its severity on an importance scale: (0) not important, (1) layout/appearance problems, (2) simple problems, (3) severe problems, and (4) catastrophic problems. After responding on the scale, they can suggest solutions.

- Efficiency

Efficiency analysis aims at the application browsability, considering the amount of effort required from users to reach the product’s goals. Hence, its approaches the visibility of elements in the application, language use, and information to do the tasks. The following questionnaire items were assessed: “Are the instructions enough to do the application’s tasks?”; “Is the usage information clear and does it help pass to other application phases?”; “Did you feel any discomfort in using the application?”. Users had three answer options for each item: “yes”; “partly”, and “no”. After the collection, data were descriptively analyzed.

- Satisfaction

Methods that measure user satisfaction with questionnaires help widely assess various types of products and systems. SUS, developed by John Brooke in 1996, is a much-known and used usability numerical scale that assesses the effectiveness, efficiency, and satisfaction of software, products, services, websites, and other types of interfaces. SUS is widely used for balancing scientific accuracy and objectivity in 10 questions^(5)^.

Data collected with SUS analyzed the responses to the 10 questions in two sets of independent data addressing two factors - Usability (eight questions) and Apprehensibility (2 questions)^(5)^. Agreement levels in the questionnaire are recorded with a 5-point Likert scale with the following 10 agreement/disagreement indications: I strongly/totally disagree (1), I disagree (2), neutral (3) I agree (4), and I strongly or totally agree (5).

Odd alternatives (items 1, 3, 5, 7, and 9) in the questionnaires are positively written about the product, while even ones (items 2, 4, 6, 8, and 10) are negative. Some terms can be adapted to the context, users, and product being assessed. After indicating each question’s agreement level, the final SUS score is obtained as follows: in odd items, 1 is subtracted from the user’s score, and in odd ones, 5 is subtracted from their response. Then, the values of the 10 questions are added and multiplied by 2.5, reaching a score that can range from 0 to 100. The assessment considers the whole questionnaire, rather than individual items, representing a compound measure of the overall system capacity^(6)^.

The author of the method does not indicate precisely what the scores represent in terms of system usability quality. However, studies conducted in different applications indicate that the mean SUS score is around 70 points and that results below this value represent serious usability problems. Other studies conducted with SUS consider this mean as a satisfactory usability index^(6)^.

NPS assessment is based on the following question: “How much would you recommend this application to someone?”. The answer to this question is indicated on a scale ranging from 0 to 10. Response-based calculations and analyses are divided into three categories: Promotors, for respondents who score 9 or 10, are satisfied and encourage people to use the application; Neutral, for those who score 7 or 8 and do not help or hinder making the application known; and Detractors, for those who score 0 to 6 as a sign of their dissatisfaction. The overall satisfaction is calculated by subtracting the percentage of promoters from the percentage of detractors. Excellent NPS results are those from 75 to 100%; very good, from 50 to 74%; average, from 0 to 49; poor from -100 to -1.

### Characterization of the sample

The study population comprised tinnitus patients attended at the Tinnitus Outreach Project of the Speech-Language-Hearing Sciences program at the Federal University of Paraiba. The sample size was calculated with G*Power software, resulting in 47 patients to make up the virtual project guidance group, although the final sample had 48 patients - 62.5% (30) were women, and 37.5% (18) were men. Their ages ranged from 25 to 65 years, with a mean age of 43.5 years and a standard deviation of 11.8. Regarding educational attainment, 8.33% (4) never went to school; 20.8% (10) and not finished elementary school; 20.8% (10) had not finished high school; 22.91% (11) were high school graduates, and 25% (12) had a bachelor’s degree.

As for participation criteria, the study included all tinnitus patients who attended the Tinnitus Outreach Project of the Speech-Language-Hearing Sciences program at the Federal University of Paraiba who had access to their own or a relative’s mobile phone with an Android operating system.

### Procedures

The research was conducted online between October and December 2021. The usability test had three main stages: first, users used the product being tested; then, they answered the usability questionnaire on their satisfaction, effectiveness, and efficiency regarding the product; lastly, the researchers analyzed the results.

Thus, the research began by inviting volunteer participants, to whom the procedures were explained. Those who agreed to participate in the research signed an informed consent form, which was sent online via Google Forms. After this stage, participants received instructions to access and use Avazum. Then, they used the application and its functions (i.e., assessed their tinnitus) and received necessary referrals, hints, and guidance on tinnitus care. Lastly, participants answered the usability questionnaire to collect data. The stages are listed separately below.

1. Inviting volunteer participants.
2. Signing the informed consent form.
3. Sending instructions to access and use the application, with explanatory images and texts.
4. Users used the Avazum application.
5. Participants answered the usability questionnaire in Google Forms.
6. Researchers analyzed the results.

### Data analysis

Data were tabulated in 2019 Microsoft Excel (16.0) for quantitative analysis. The Statistical Package for the Social Sciences (SPSS), version 21.0.4.7, was used for the descriptive analysis, addressing frequency, percentages, and SUS score analysis.

### Ethical considerations

This project was submitted to and approved by the Research Ethics Committee of the Department of Health Sciences of the Federal University of Paraiba, under evaluation report number 4.297.792. Informed consent was obtained from all participants. All procedures complied with the guidelines and parameters of the agencies that regulate health ethics norms.

## RESULTS

Avazum was analyzed regarding its efficiency and effectiveness and participating user satisfaction. They analyzed aspects such as visibility of elements in the application, language use, information to do the tasks, assessments, guidance, and referrals of tinnitus patients. The results are presented below.

### Satisfaction

This item analyzed 10 SUS questions and the NPS assessment, in which users scored their level of recommendation of the application, ranging from 0 to 10. The overall SUS score was 78.28, considered a good score that demonstrates the absence of severe usability problems. Most users reported they would like to use the application often and could easily use it, denying the need for support to use the Avazum functions well - despite the presence of older participants and people with low educational attainment in the sample. These results can be seen in detail in Table 1. Avazum was found to be consistent, and its functions are well-integrated, providing comfort and confidence in its use, as seen in the results in Table 2.

**Table 1:** Questions related to the satisfaction with Avazum application

		**I would like to use the application often**		**I found the application unnecessarily complicated**	
		Frequency	Percentage	Frequency	Percentage
	I totally disagree	0	0	12	25
	I disagree	3	6.3	33	68.8
	Neutral	12	25	3	6.3
	I agree	23	47.9	0	0
	I totally agree	10	20.8	0	0
Total		48	100	48	100

**Source:** The authors

**Table 2:** Questions related to the ease of using the Avazum application

		**I found the various application functions well integrated**		**I found many inconsistencies in the application**		**I felt very confident using the application**	
		Frequency	Percentage	Frequency	Percentage	Frequency	Percentage
	I totally disagree	0	0	12	25	0	0
	I disagree	0	0	33	68.8	2	4.2
	Neutral	7	14	2	4.2	7	14.6
	I agree	33	68.8	1	2.1	29	60.4
	I totally agree	8	16.7	0	0	10	20.8
Total		48	100	48	100	48	100

**Figure 1:**
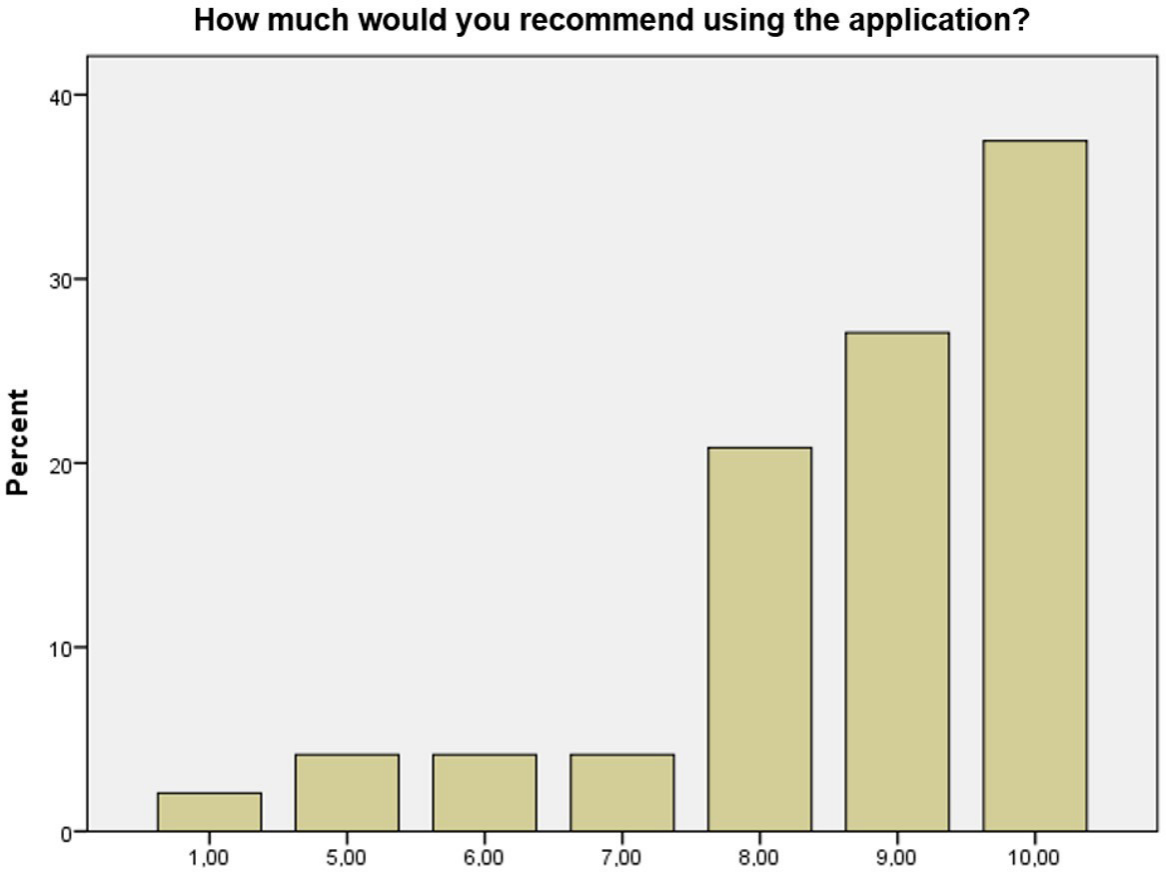
Percentages of user satisfaction

Concerning the key satisfaction points, participants were satisfied and encouraged others to use the application. Thus, the NPS scale and SUS score verified good user satisfaction regarding Avazum. The overall NPS percentage was 58%, indicating that NPS was very good - i.e., there was a higher satisfaction rate. Values are shown in detail in Figure 1, below.

Caption: The figure above shows the percentages of user satisfaction with Avazum, based on the question “How much would you recommend using the application?”. The horizontal line presents scores ranging from and 0 to 10, the vertical line has the percentages of the users’ scores. **Source:** The authors

### Effectiveness

Users understood well the tasks, instructions, and language in the application. They followed all tasks and described their tinnitus using the application. The hints and guidance provided in Avazum proved to be relevant/interesting to their realities. As for referrals to professionals. They were disappointed with the lack of clarity. Therefore, referrals must be clearer, and the list of professionals must be more complete. The list of professionals was updated based on a master’s research on the “profile of professionals specialized in treating tinnitus patients in Brazil” and will be added to the application in its next update. Tables 3 and 4 show the results in further detail.

**Table 3:** Questions related to Avazum application efficiency

		**Did you understand the instructions in the application? Was the language clear?**		**Could you describe your tinnitus?**	
		Frequency	Percentage	Frequency	Percentage
					
	Yes	36	75	40	83.3
	Partly	11	22.9	8	6.3
	No	1	2.1	0	0
	Total	48	100	48	100

**Source:** The authors

**Table 4:** Questions related to Avazum application effectiveness

		**Were the instructions regarding tinnitus in the application relevant/interesting to your reality?**		**Was the referral to healthcare with professionals objective and easy to access?**	
		Frequency	Percentage	Frequency	Percentage

	Yes	47	97.9	20	41.7
	Partly	1	2.1	26	54.2
	No	0	0	2	4.2
	Total	48	100	48	100

**Source:** The authors

### Efficiency

Concerning application efficiency, the assessments were sufficient for the key application goals, and the information on the screens was clear - thus, browsing Avazum was comfortable. Table 5 shows the information in further detail.

**Table 5:** Questions related to the Avazum application efficiency

		**Were the instructions enough to do the application tasks?**		**Is usage information clear, helping pass to subsequent application phases?**		**Did you feel any discomfort in using the application?**	
		Frequency	Percentage	Frequency	Percentage	Frequency	Percentage
							
	Yes	45	93.8	46	95.8	8	16.7
	Partly	3	6.3	2	4.2	1	2.1
	No	0	0	0	0	39	81.3
	Total	48	100	48	100	48	100

Thus, after analyzing the SUS score, its mean proved to be satisfactory, which indicates that the application has no severe usability problems and a good percentage of promoting users, who scored 9 and 10 in NPS satisfaction. The overall NPS percentage showed that the user satisfaction is very good. Moreover, the data descriptive analysis showed that Avazum effectively reached its goals, assessing tinnitus, making referrals, and providing hints and guidance to users. It is also efficient, as its language is clear, and its use is comfortable. However, the effectiveness item that assessed referrals was flawed regarding its ease of use and instruction on access.

## DISCUSSION

Usability is an important aspect of the quality of products and systems. It is a concept widely used in product development, aiming to observe people using the product, based on the interaction between the human, the task, and the product. A growing number of companies are recognizing not only the importance of usability in the design process but also its potential to ensure advantages in the market. Given such importance, the topic has been addressed in various studies by researchers and specialists^(7)^. The main focus of the usability test is the users’ needs, aiming to provide them with an excellent experience. Thus, the essential focus of usability evidently is the ease of use when interacting with the product.

Usability tests count on tools and questionnaires to verify the effectiveness, efficiency, and satisfaction with the tested product. According to ISO 9241:11 (1998), effectiveness is normally assessed by the number of stages the user successfully completed, efficiency focuses on the time users append to reach the goals, and satisfaction is assessed with protocols. User satisfaction is directly related to the levels of comfort and enjoyment in using the product^(8)^. Methods that measure user satisfaction with questionnaires contribute to a comprehensive assessment of various types of products and systems^(8)^.

Usability is a key tool to develop health technology innovation tools. Hence, this stage must be carefully carried out, focusing on real users. A study aimed to verify the methodology of usability tests regarding health applications and showed that 75.9% of them were tested in real users, such as patients and professionals, whereas only 6% were tested in specialized professionals. It also demonstrated that most tests used quantitative and qualitative questionnaires^(9)^.

SUS score ranges from 0 to 100, although the questionnaire validation study does not provide details of what each score represents regarding a product’s usability. However, the study by Zorzal (2009) demonstrates that adequate scores range from 70 to 100, below which, the product may have serious usability problems. Hence, mean scores above 70 are satisfactory, showing that Avazum did not have major usability problems.

Satisfaction was also analyzed based on NPS, which is widely used to verify clients’ satisfaction with products, services, attention, and so on. When the percentage of promoting clients/users is greater than that of detractors, the product is more likely to be indicated by people who use it. As for neutrals or passive clients, they are not dissatisfied but are unlikely to indicate it to other people. A total NPS score above 50% shows a very good satisfaction with the product, whereas, below 49%, the satisfaction was not good^(10)^.

To obtain high usability, an application must have adequate effectiveness and efficiency levels. Characteristics such as good menu accessibility, common functions in the screens, fonts and sizes that favor reading even among users with difficulties, and simple tasks that make the next step clear are important to efficiency assessment. Hence, an application with clear language, enough instructions to do tasks, and clear information to pass to other screens is efficient regarding its functions^(11)^.

Effective products and services can reach their goals and meet users’ real needs. The activities in the final version of mobile applications must be functional, considering whether it has enough functions. Usability tests must analyze their efficiency considering each item and the functions that did not meet the users’ needs^(12)^.

A study assessed 66 applications available to people with diabetes, using a 5-point Likert scale. The results showed mean answers between 3 and 4, indicating that applications had moderate to good usability, especially the ones that were easy to use and had clear language^(13)^.

As a limitation of the study, it is important to mention that Avazum is not yet available in the iOS system. Hence, the usability test could not include patients who use this mobile system. The next steps will include the application for the iOS system, translating and adapting it to other languages, and conducting further tests encompassing the updates.

## CONCLUSION

Users understood all tasks and described their tinnitus using the application, thus demonstrating that Avazum was effective regarding its goals. Also, the application functions are well integrated, clearly arranged, and comfortable to use, proving to efficiently do its functions. Moreover, Avazum users presented a good satisfaction index. It is a promising application to help assess tinnitus, refer users, and promote health through its instructions.

## Annex A. Usability Questionnaire

Satisfaction

1.1 System Usability Scale (SUS) (BROOKE, 1996)

Regarding your satisfaction with the application, please answer:

1 I totally disagree

2 I disagree

3 Neutral

4 I agree

5. I totally agree

1. I would like to use this application often

2. I found the application unnecessarily complicated

3. I found the application easy to use

4. I thought I would need support from a more specialized person to use this application

5. I thought the various application functions are well integrated

6. I found many inconsistencies in this application

7. I imagine most people would quickly learn how to use this application

8. I felt very confident using this application

9. I would need to learn many things before I could use this application

1.2 Net Promoter Score

How much would you recommend this application to someone?

() 0 ()1 ()2 ()3 ()4 ()5 () 6 ()7 ()8 ()9 ()10

Use effectiveness

2.1 When you used the application, did you understand its tasks and instructions? Is its language clear?

[ ] Yes, I understood all of them

[ ] Yes, I understood some of them

[ ] No, I didn’t understand them

If you answered one of the last two alternatives, please indicate how serious the problem was:

0 - Not important

1 - Layout/appearance problem

2 - Simple problem

3 - Severe problem

4 - Catastrophic problem

Do you have any suggestions to fix the problem?

2.2 Could you describe your tinnitus by answering the application questions?

[ ] Yes

[ ] Partly

[ ] No

If you answered one of the last two alternatives, please indicate how serious the problem was:

0 - Not important

1 - Layout/appearance problem

2 - Simple problem

3 - Severe problem

4 - Catastrophic problem

Do you have any suggestions to fix the problem?

2.3 Were the instructions in the tinnitus application relevant/interesting to your reality?

[ ] Yes

[ ] Partly

[ ] No

If you answered one of the last two alternatives, please indicate how serious the problem was:

0 - Not important

1 - Layout/appearance problem

2 - Simple problem

3 - Severe problem

4 - Catastrophic problem

Do you have any suggestions to fix the problem?

2.4 Was the referral to healthcare with professionals objective and easy to access?

[ ] Yes

[ ] Partly

[ ] No

If you answered one of the last two alternatives, please indicate how serious the problem was:

0 - Not important

1 - Layout/appearance problem

2 - Simple problem

3 - Severe problem

4 - Catastrophic problem

Do you have any suggestions to fix the problem?

Use efficiency

3.1 Visibility of elements in the application

The visual arrangement of images is adequate

[ ] Yes

[ ] Partly

[ ] No

If you answered one of the last two alternatives, please indicate how serious the problem was:

0 - Not important

1 - Layout/appearance problem

2 - Simple problem

3 - Severe problem

4 - Catastrophic problem

Do you have any suggestions to fix the problem?

Font sizes are adequate and readable

[ ] Yes

[ ] Partly

[ ] No

If you answered one of the last two alternatives, please indicate how serious the problem was:

0 - Not important

1 - Layout/appearance problem

2 - Simple problem

3 - Severe problem

4 - Catastrophic problem

Do you have any suggestions to fix the problem?

3.2 Language used

Instructions are enough to do the application tasks

[ ] Yes

[ ] Partly

[ ] No

If you answered one of the last two alternatives, please indicate how serious the problem was:

0 - Not important

1 - Layout/appearance problem

2 - Simple problem

3 - Severe problem

4 - Catastrophic problem

Do you have any suggestions to fix the problem?

Usage information is clear and helps pass to subsequent application phases

[ ] Yes

[ ] Partly

[ ] No

If you answered one of the last two alternatives, please indicate how serious the problem was:

0 - Not important

1 - Layout/appearance problem

2 - Simple problem

3 - Severe problem

4 - Catastrophic problem

Do you have any suggestions to fix the problem?

3.4 Comfort

Did you feel any discomfort in using the application

[ ] Yes

[ ] Partly

[ ] No

If you answered one of the last two alternatives, please indicate how serious the problem was:

0 - Not important

1 - Layout/appearance problem

2 - Simple problem

3 - Severe problem

4 - Catastrophic problem

Do you have any suggestions to fix the problem?
