# Supplementary material for: Avazum app usability testing
Source: Codas. 2023 Oct 9;35(5):e20220103. doi: 10.1590/2317-1782/20232022103en (PMC10688289; doi:10.1590/2317-1782/20232022103en)
Supplement: Translation

# Translation: Teste de usabilidade do aplicativo Avazum

**DOI:** 10.1590/2317-1782/20232022103pt

**Authors:** Hionara Nascimento Barboza, Marcillyo Carneiro de Lima, Rubens Jonatha dos Santos Ferreira, Marine Raquel Diniz da Rosa, Ana Loisa de Lima e Silva Araújo, Angélica de Souza Galdino Acioly

**Affiliations:** 1 Departamento de Fonoaudiologia, Universidade Federal da Paraíba - UFPB - João Pessoa (PB), Brasil.; 2 Departamento de Design, Universidade Federal da Paraíba - UFPB - João Pessoa (PB), Brasil.

**Keywords:** Aplicativos Móveis, Zumbido, Estudo de Avaliação, Design Centrado no Usuário, Projetos de Desenvolvimento Tecnológico e Inovação

## Abstract

**Objetivo:**

Verificar a efetividade, eficiência e satisfação no teste de usabilidade para o aplicativo de avaliação do zumbido.

**Método:**

Trata-se de um estudo descritivo de teste de usabilidade, no qual será avaliado satisfação, eficácia e eficiência do aplicativo. O teste foi realizado virtualmente por meio de um formulário do Google Forms. Primeiramente, os participantes receberam as instruções de acesso e uso do avazum através de textos e imagens ilustrativas. Logo após, os usuários fizeram o uso do aplicativo, observando suas funções e usabilidade. Em seguida, os participantes responderam o questionário de usabilidade. A análise estatística foi realizada no software Statistics Package for the Social Sciences (SPSS) versão 21.0 para realizar a análise descritiva como frequência, percentagens dos dados do System Usability Questionnaire (SUS), além de análise do score do Net Promoter Score (NPS).

**Resultados:**

Sendo assim, a média geral do score do SUS foi condizente com score de usabilidade satisfatório, o que implica dizer que o aplicativo não apresenta graves problemas de usabilidade, além da porcentagem geral NPS indicar uma satisfação muito boa dos usuários, com bom percentual de usuários promotores. No que diz respeito sobre a efetividade, foi possível observar que o Avazum atinge os objetivos propostos, além de ser eficiente, pois apresenta uma linguagem clara e confortabilidade durante o uso.

**Conclusão:**

O Avazum obteve satisfação muito boa dos usuários, além de atingir os objetivos propostos, tornando-o efetivo, apresentou uma linguagem clara e confortabilidade durante o uso, aduzindo eficiência na avaliação multidisciplinar do zumbido.

## INTRODUÇÃO

A revolução científica vem trazendo à sociedade um olhar mais amplo ao lidar com o mundo, proporcionando avanços em todas as áreas, considerando que a tecnologia é um fruto da ciência moderna que uniu técnicas e métodos para desenvolvê-la e fazer dela a potência e instrumento que é hoje^(1)^. No campo da saúde, a tecnologia tornou-se uma grande aliada com avanços em procedimentos, em técnicas, curas, inovação em educação em saúde, atendimentos e dentre outros. O rápido desenvolvimento e uso generalizado de tecnologias móveis vêm expandindo novas oportunidades de atividades em saúde, uma nova era se faz presente, a *mobile health* (*mHealth*), mais conhecida como saúde móvel^(2)^.

Na Fonoaudiologia, a inovação tecnológica está se tornando umas das principais aliadas em todas suas áreas de atuação, seja em processos, produtos ou serviços em saúde. Atualmente, vários aplicativos e *softwares* estão sendo desenvolvidos para inovações na execução de terapias, avaliação, educação em saúde e dentre outros. No que se refere ao zumbido, hoje estão disponíveis uma diversidade de aplicativos que auxiliam no tratamento, como terapia sonora e terapia cognitivo comportamental. Os aplicativos com maiores leques de disponibilidades nas plataformas são os com ênfase em terapia, poucos tem funções voltadas para avaliação multidisciplinar que contém funções de encaminhamento diferencial. É importante mencionar que a maior parte dos aplicativos não realizou o teste de usabilidade^(3)^.

Nesse sentido, O Avazum é um aplicativo desenvolvido para auxiliar na avaliação inicial do zumbido, ajudando na triagem e encaminhamento dos usuários para os profissionais necessários, realizando uma avaliação detalhada, segundo a necessidade de cada caso, além de disponibilizar dicas e orientações sobre os cuidados com o zumbido. O aplicativo apresenta três interfaces principais, a tela de cadastro, as telas para avaliação propriamente dita e as telas de resultados, encaminhamentos, dicas e orientações. Foi desenvolvido por uma equipe interdisciplinar entre Fonoaudiólogos, Programador e Designers, membros de dois grupos de pesquisa em inovação tecnológica em saúde e grupo de estudo e pesquisa em zumbido da Universidade Federal da Paraíba. O aplicativo já passou por todas as fases de desenvolvimento, sendo realizado o teste de usabilidade no presente estudo.

Teste de usabilidade é um método de verificação de funcionalidades da interface de uma plataforma digital. É empregado em websites, aplicações e outras ferramentas, levando usuários reais à execução de determinadas tarefas. Após seu desenvolvimento, é realizada uma análise de usabilidade e das principais dificuldades. Por meio de testes de usabilidade, pode-se registrar os melhores resultados obtidos para futuras atualizações levando à minimização do custo do serviço de suporte aos usuários, crescimento de vendas e prever o lançamento de produtos com menos problemas de usabilidade.

O teste de usabilidade é um quesito imprescindível no desenvolvimento de um produto com inovação tecnológica, pois analisa as informações necessárias para detectar eventuais problemas de usabilidade e consequentemente, fornece ferramentas para entregar um produto com qualidade para os usuários^(4)^.

Na área da saúde, a prática baseada em evidência é essencial para oferecer um serviço de excelência com resultados comprovados. Ao desenvolver um produto de inovação para essa área, é imprescindível buscar a compreensão das necessidades do usuário para que se possa atender com objetividade e qualidade a experiência desejada na realização das funções alvo.

Dessa forma, o objetivo deste estudo é verificar a efetividade, eficiência e satisfação no teste de usabilidade para o aplicativo de avaliação do zumbido.

## MÉTODO

### Caracterização do Avazum

O aplicativo contém inicialmente uma tela de cadastro do usuário com e-mail e senha para realizar o *login*. Após o cadastro, os usuários serão direcionados para as telas de avaliação, com todos os aspectos relacionados ao sintoma, como ocorreu seu início, caracterização do tipo de som e localização de forma interativa, com recursos áudios visuais. Posteriormente, terão as sessões sobre os hábitos que pioram ou melhoram a percepção do zumbido, e mensuração do incômodo com o auxílio da Escala Visual Analógica. Após todas as etapas de avaliação concluídas, os usuários terão acesso ao resultado, indicando os possíveis sintomas e causas associadas. Além, da indicação para os profissionais de acordo com as especificidades de cada caso avaliado, baseados nos descritores de encaminhamento, relacionando os sintomas descritos com os profissionais específicos de cada área. Os profissionais que estão inseridos são: Fonoaudiólogo, Otorrinolaringologista, Psicólogo, Fisioterapeuta e Nutricionista. Ao final, ficarão disponibilizados dicas e orientações sobre os cuidados do o zumbido, relacionados com saúde física e mental. O número de registro do aplicativo no INPI é BR512020001425-9. Mais detalhes sobre o aplicativo ficarão disponíveis em um outro estudo intitulado “Desenvolvendo o Avazum: Aplicativo de avaliação interativa do zumbido” no qual será publicado.

### Desenho do estudo e instrumentos utilizados

Estudo descritivo de teste de usabilidade do Aplicativo de Avaliação do Zumbido (Avazum).

Os instrumentos utilizados para pesquisa de usabilidade contemplaram as dimensões de satisfação, eficiência e efetividade do aplicativo (Anexo A), sendo a satisfação avaliada por método empírico, através do questionário de usabilidade *System Usability Questionnaire* - SUS e do *Net Promoter Score* - NPS e as dimensões de eficiência e eficácia de uso, por método analítico, considerando princípios heurísticos para o aplicativo.

- Efetividade

A avaliação foi direcionada à coleta de informações sobre o alcance dos objetivos por parte dos usuários e a capacidade do produto para fazer o que se propõe. Nesse propósito, são abordados os aspectos de avaliação, orientações e encaminhamentos de pacientes com zumbido. Nas dimensões de eficiência e efetividade, as respostas são observadas a partir da presença ou ausência de problemáticas a serem solucionadas. No caso de apontamento afirmativo para o problema, o usuário apontará a severidade na escala de importância: (0) sem importância, (1) problema cosmético/aparência, (2) problema simples, (3) problema grave e (4) problema catastrófico. Após responder na escala, realizará a sugestão de correção.

- Eficiência

Na eficiência, a análise é direcionada à navegabilidade do aplicativo, considerando a quantidade de esforço exigida dos usuários para atingir os objetivos propostos pelo produto. Nesse intuito, são abordados a visibilidade dos elementos do aplicativo, utilização da linguagem, informações para realização das tarefas. Os itens do questionário que foram avaliados na eficiência foram “Os enunciados são suficientes para realização das tarefas propostas no aplicativo?”; As informações de uso são claras e possibilitam a passagem de fases no aplicativo?”; “Você sentiu algum desconforto na utilização do aplicativo?”. Para cada item os usuários tinham três opções “sim”; “em parte” e “não”. Após a coleta, foi realizada a análise descritiva dos dados.

- Satisfação

Métodos que permitem medir a satisfação dos usuários por meio de questionários contribuem para uma avaliação ampla para vários tipos de produtos e sistemas. Um questionário conhecido e muito utilizado é o SUS- *System Usability Scale*, por John Brooke em 1986, é uma escala numérica de usabilidade que efetividade, eficiência e satisfação de 10 software, produtos, serviços, websites e outros tipos de interface. O SUS é bastante utilizado por ter um equilíbrio em ser cientificamente apurado e objetivo, consiste em 10 perguntas^(5)^.

A análise dos dados coletados com o questionário de usabilidade *System Usability Questionnaire* - SUS foram verificados a partir das respostas das dez declarações em dois conjuntos de dados independentes através de dois fatores - Usabilidade (8 questões) e Apreensibilidade (2 questões)^(5)^. Para o registro dos níveis de concordância, o questionário utiliza uma escala de Likert de 5 pontos com as seguintes 10 indicações de concordância/discordância: discordo fortemente ou totalmente (1), discordo (2), uma de neutralidade (3) e duas de concordância: concordo (4) e concordo fortemente ou totalmente (5).

As alternativas ímpares do questionário são redigidas de forma positiva sobre o produto avaliado, ou seja, os itens 1,3,5,7 e 9, enquanto os pares de forma negativa, itens 2,4,6,8 e 10. Tendo a possibilidade de alguns termos usados serem adaptados ao contexto, usuário e produto avaliado. Para obter o score final do SUS, após a indicação dos níveis de concordância de cada questão, para as respostas ímpares, subtrai-se 1 da pontuação que o usuário respondeu, para as respostas pares subtrai-se 5. Em seguida, os valores obtidos para as dez perguntas serão somados e multiplicados por 2,5, essa pontuação pode ir de 0 a 100, representando uma medida composta da capacidade geral do sistema, sendo a avaliação em conjunto, não por itens individuais^(6)^.

O autor do método não apresenta precisamente o que a pontuação obtida pode representar em termos de qualidade da usabilidade do sistema avaliado, contudo, estudos realizados em diferentes aplicações indicam que a média do SUS gira em torno de 70 pontos, e que resultados abaixo desse valor representam problemas sérios de usabilidade^(6)^.

Para o NPS, a avaliação é baseada na pergunta: O quanto você recomendaria este aplicativo para alguém? A resposta para pergunta será evidenciada em escala de 0 a 10. O cálculo e análise com base na resposta são divididos em três categorias: Promotores, para os respondentes que dão as notas 9 ou 10, estão satisfeitos e incentivam pessoas ao uso do aplicativo; Neutros para os respondentes que dão as notas 7 ou 8, não ajudam a divulgar o aplicativo, mas não atrapalham; e Detratores, para os respondentes que dão as notas de 0 a 6, como evidência de insatisfação. O cálculo para verificar a satisfação geral é realizado pela subtração da porcentagem dos promotores pela porcentagem de detratores. Resultado de 75 a 100% é considerado NPS excelente; 50 a 74%-NPS muito bom; 0 a 49- NPS razoável; -100 a -1 NPS ruim.

### Caracterização da amostra

A população do estudo foi composta por pacientes com zumbido atendidos no Projeto de Extensão de Zumbido, do curso de Fonoaudiologia da Universidade Federal da Paraíba. Foi realizado o cálculo amostral pelo o software G*Power no qual resultou em 47 pacientes, que compõem o grupo virtual de orientação do projeto, porém a amostra final foi composta por 48 pacientes ao total. Dentre os 48 pacientes, 62,5% (30) foram mulheres e 37,5% (18) homens. As idades dos participantes da pesquisa foram de 25 a 65 anos, com idade média de 43,5 anos e desvio padrão de 11,8. Com relação à escolaridade, 8,33% (4) nunca foram à escola; 20,8% (10) ensino fundamental incompleto; 20,8% (10) ensino médio incompleto; 22,91% (11) ensino médio completo e 25% (12) ensino superior completo.

No que se refere aos critérios de participação, foram incluídos todos os pacientes que possuíam zumbido, eram atendidos no Projeto de Extensão de Zumbido, do curso de Fonoaudiologia da Universidade Federal da Paraíba e que tinham acesso ao um celular de uso próprio ou de algum familiar com sistema *android*.

### Procedimentos

A pesquisa foi realizada de forma virtual no período de outubro a dezembro de 2021. O teste de usabilidade foi realizado em 3 etapas principais: primeiro os usuários utilizaram o produto alvo do teste; em seguida responderam aos questionários de usabilidade sobre a sua satisfação, eficácia e eficiência do produto testado; e por último, realizou-se a análise dos resultados pelos pesquisadores.

Sendo assim, a seguinte pesquisa teve início com o convite para a participação voluntária com explicação dos seus procedimentos, após o aceite para participação da pesquisa e assinatura do Termo de Consentimento Livre e Esclarecido- TCLE, o qual foi enviado virtualmente como um formulário do *Google Forms*. Cumprida essa etapa, os participantes receberam as instruções de acesso e uso do Avazum, e em seguida, fizeram uso do aplicativo e suas funções, ou seja, realizaram a avaliação do zumbido, além de receberem os encaminhamentos necessários, dicas e orientações sobre os cuidados do zumbido. Por último, os participantes responderam o questionário de usabilidade para a coleta de dados. Abaixo estão listadas as etapas separadamente.

1.Convite para participação voluntária;
2.Assinatura do TCLE;
  1. Envio das instruções de acesso e uso do aplicativo através de imagens e textos explicativos;
  2. Os usuários fizeram o uso do aplicativo Avazum;
  3. Os participantes responderam os questionários de usabilidade através do google forms;
  4. Análise dos resultados pelos pesquisadores.

### Análise dos dados

Os dados foram tabulados no programa Microsoft Excel 2019 (16.0), o tipo de análise dos dados foi realizado de forma quantitativa. Foi utilizado o software *Statistics Package for the Social Sciences* (SPSS) versão 21.0. 4.7 para realizar a análise descritiva como frequência, percentagens, além de análise do escore do SUS.

### Considerações éticas

Este projeto foi submetido e aprovado pelo Comitê de Ética em Pesquisa do Centro de Ciências da Saúde da Universidade Federal da Paraíba, com número do parecer de aprovação 4.297.792. Consentimento informado foi obtido de todos os participantes. Todos os procedimentos ocorreram de acordo com as diretrizes e balizamentos dos órgãos que regem as normativas éticas em saúde.

## RESULTADOS

O Avazum foi analisado nos aspectos de eficiência, efetividade e satisfação dos usuários participantes. Os pontos analisados foram desde visibilidade dos elementos do aplicativo, utilização da linguagem, informações para realização das tarefas, como também, aspectos de avaliação, orientações e encaminhamentos de pacientes com zumbido. Sendo assim, os resultados serão apresentados abaixo.

### Satisfação

Nesse quesito foram analisadas 10 declarações do questionário SUS, além da avaliação do NPS, no qual os usuários deram a nota de recomendação dos aplicativos, variando de 0 a 10. A média geral do score do SUS foi 78,28, evidenciando que não há problemas graves de usabilidade, sendo considerado um escore bom. A maior parte dos usuários evidenciaram que gostariam de utilizar o aplicativo com frequência e demonstraram facilidade em utilizá-lo, negando que precisariam de apoio para utilizar bem as funções do Avazum, mesmo havendo participantes idosos e com baixa escolaridade na amostra. É possível observar esses resultados detalhadamente nas Tabela 1. Foi possível observar que o Avazum é consistente e suas funções são bem integradas, propiciando um conforto e confiança ao utilizá-lo, como mostram os resultados na Tabela 2.

**Tabela 1:** Questões relacionadas à satisfação do aplicativo Avazum

		**Gostaria de usar o aplicativo com frequência**		**Achei o aplicativo desnecessariamente complicado**	
		Frequência	Percentual	Frequência	Percentual
	Discordo totalmente	0	0	12	25
	Discordo	3	6,3	33	68,8
	Neutro	12	25	3	6,3
	Concordo	23	47,9	0	0
	Concordo totalmente	10	20,8	0	0
Total		48	100	48	100

**Fonte:** Autoria própria

**Tabela 2:** Questões relacionadas à facilidade de uso do aplicativo Avazum

		**Achei que as diversas funções do aplicativo estão bem integradas**		**Achei que havia muita inconsistência neste aplicativo**		**Eu me senti muito confiante usando o aplicativo**	
		Frequência	Percentual	Frequência	Percentual	Frequência	Percentual
	Discordo totalmente	0	0	12	25	0	0
	Discordo	0	0	33	68,8	2	4,2
	Neutro	7	14	2	4,2	7	14,6
	Concordo	33	68,8	1	2,1	29	60,4
	Concordo totalmente	8	16,7	0	0	10	20,8
Total		48	100	48	100	48	100

Em relação ao ponto chave da satisfação, os participantes demonstraram satisfeitos e incentivadores ao uso do aplicativo. Dessa forma, foi possível constatar que houve uma boa satisfação dos usuários com o uso do Avazum, através da escala do NPS e score do SUS. Após realizar o cálculo para obter a porcentagem geral do NPS, o resultado foi de 58%, indicando que o NPS foi muito bom, ou seja, houve maiores índices de satisfação. Os valores detalhados podem ser observados no gráfico abaixo representado na Figura 1.

**Figura 1:** Porcentagens sobre a satisfação dos usuário

Legenda: A figura acima apresenta as porcentagens da satisfação dos usuários ao utilizar o Avazum, a partir da pergunta “O quanto recomendaria o uso do aplicativo?”. Na linha horizontal estão dispostas as notas variando de 0 a 10. Na linha vertical, estão dispostos os números em porcentagens das notas dos usuários. **Fonte:** Autoria própria

### Efetividade

Foi possível observar que houve boa compreensão dos usuários no que se refere às tarefas e orientações apresentadas pelo aplicativo e a linguagem utilizada. Demonstraram compreender todas as tarefas e conseguiram descrever o zumbido utilizando o aplicativo. Com relação às dicas e orientações oferecidas no Avazum, demonstraram ser relevantes/interessantes para suas realidades. Ao serem questionados sobre os encaminhamentos para os profissionais, houve desapontamentos sobre a clareza dos direcionamentos. Sendo assim, os encaminhamentos precisam ser mais claros e a lista dos profissionais ser mais completa. É importante mencionar que a lista dos profissionais foi atualizada com base em uma pesquisa de mestrado sobre “Perfil dos Profissionais Especializados no Atendimento em Pacientes com Zumbido no Brasil” será adicionada na próxima atualização do *app*. Nas Tabelas 3 e 4 os resultados podem ser observados com mais detalhes.

**Tabela 3:** Questões relacionadas com eficácia do aplicativo Avazum

		**Compreendeu as tarefas e orientações apresentadas pelo aplicativo? A linguagem é clara?**		**Conseguiu descrever o zumbido?**	
		Frequência	Percentual	Frequência	Percentual
					
	Sim	36	75	40	83,3
	Em parte	11	22,9	8	6,3
	Não	1	2,1	0	0
	Total	48	100	48	100

**Fonte:** Autoria própria

**Tabela 4:** Questões relacionadas com eficácia do aplicativo Avazum

		**As orientações no aplicativo sobre zumbido foram relevantes/interessantes para sua realidade?**		**O encaminhamento para seguimento do atendimento com profissionais foi direcionado e de fácil acesso?**	
		Frequência	Percentual	Frequência	Percentual

	Sim	47	97,9	20	41,7
	Em parte	1	2,1	26	54,2
	Não	0	0	2	4,2
	Total	48	100	48	100

**Fonte:** Autoria própria

### Eficiência

No que diz respeito à eficiência do aplicativo, foi possível observar que as avaliações foram suficientes para os objetivos chaves do *app,* além das informações sobre as telas serem apresentadas com clareza, consequentemente, apresentando conforto ao navegar no Avazum. Na Tabela 5 as informações podem ser visualizadas com mais minuciosidade.

**Tabela 5:** Questões relacionadas à eficiência do aplicativo Avazum

		**Os enunciados são suficientes para realização das tarefas propostas no aplicativo?**		**As informações de uso são claras e possibilitam a passagem de fases no aplicativo?**		**Você sentiu algum desconforto na utilização do aplicativo?**	
		Frequência	Percentual	Frequência	Percentual	Frequência	Percentual
							
	Sim	45	93,8	46	95,8	8	16,7
	Em parte	3	6,3	2	4,2	1	2,1
	Não	0	0	0	0	39	81,3
	Total	48	100	48	100	48	100

Dessa forma, após a realização da análise realizada do escore do questionário SUS, a média encontrada é condizente com uma média de score satisfatória, o que implica dizer que o aplicativo não apresenta graves problemas de usabilidade, além de um bom percentual de usuários promotores, ou seja, deram notas 9 e 10 para satisfação no NPS. A porcentagem geral do NPS mostrou que a satisfação dos usuários é muito boa, já em relação à efetividade, após a análise descritiva dos dados, foi possível observar que o Avazum atinge os objetivos propostos, consegue realizar avaliação do zumbido, os encaminhamentos e oferecer dicas e orientações aos usuários. Além de ser eficiente, pois apresenta uma linguagem clara e confortabilidade durante o uso. É importante mencionar que o item avaliado na eficácia referente aos encaminhamentos dos usuários, apresentou um déficit sobre a facilidade e direcionamento do acesso.

## DISCUSSÃO

A usabilidade é um quesito importante para a qualidade de produtos e sistemas. Consiste em um conceito amplamente utilizado no desenvolvimento de produtos, tem como objetivo principal observar as pessoas usando um produto, a partir da interação humano, tarefa e produto. Um número crescente de empresas está reconhecendo não só a importância da usabilidade no processo de design, mas também o seu potencial para garantir vantagens no mercado. Devido a esta importância, o tema tem sido alvo de vários estudos propostos por pesquisadores e especialistas^(7)^. O teste de usabilidade tem foco principal nas necessidades dos usuários, concentrando-se em trazer uma excelente experiência para os mesmos. Dessa forma, fica evidente que o foco fundamental da usabilidade continua sendo a facilidade de uso quando interage com o produto.

Os testes de usabilidade são realizados com auxílio de ferramentas e questionários para verificar a efetividade, eficiência e satisfação com o produto testado. Segundo a ISO9241:11 (1998), a efetividade normalmente é avaliada pela quantidade de etapas que o usuário conseguiu completar com sucesso, a eficiência tem foco no tempo gasto para cumprir os objetivos, já a satisfação é avaliada por meio de protocolos. A satisfação dos usuários tem relação direta com os níveis de conforto ao usar o produto, como também o quanto gostou da utilização da ferramenta em questão^(8)^. Métodos que permitem medir a satisfação dos usuários por meio de questionários contribuem para uma avaliação ampla para vários tipos de produtos e sistemas^(8)^.

A usabilidade é ferramenta chave para o desenvolvimento de produtos de inovação tecnológica em saúde, uma etapa que deve ser realizada com muita atenção e direcionada para usuários reais. Em um estudo direcionado para verificar a metodologia dos testes de usabilidade nos aplicativos em saúde, mostrou que 75,9% foram testados em usuários reais, como pacientes e profissionais e apenas 6% testados com profissionais especializados. Além de evidenciar que a maior parte dos testes foram realizados com questionários de forma quantitativa e qualitativa^(9)^.

O escore do questionário SUS varia de 0 a 100, não há detalhes no estudo de validação do questionário, sobre o que cada escore pode representar em relação à usabilidade de um produto. Porém, o estudo de Zorzal (2009) evidencia que um score adequado varia de 70 a 100, abaixo disso o produto pode apresentar problemas sérios de usabilidade, sendo assim, a média do escore a partir de 70 é satisfatória. Mostrando assim, que o Avazum não apresentou grandes problemas de usabilidade.

Outro quesito analisado foi à satisfação a partir da Net promoter score- NPS, muito utilizado para medir a satisfação dos clientes sobre produtos, serviços, atendimentos e dentre outros. Quando a porcentagem dos clientes/usuários promotores é maior que os detratores, o produto tem mais chance de ser indicado pelas pessoas que o utilizaram, já os neutros ou passivos, não estão insatisfeitos, mas provavelmente não indicarão para outras pessoas. Um score total a partir de 50% no NPS mostra que tiveram uma satisfação muito boa com o produto utilizado, mas abaixo de 49% não houve uma satisfação boa^(10)^.

Para obter uma usabilidade alta de um aplicativo, é necessário que haja níveis de efetividade e eficiência adequados para o que é proposto. Características como boa acessibilidade dos menus e funções comuns nas telas, fontes e tamanho que favorecem a leitura mesmo para aquele usuário com dificuldade, tarefas simples de serem executadas, deixando claro o próximo passo, são importantes na avaliação da eficiência. Sendo assim, um aplicativo com linguagem clara, enunciados suficientes para realização das tarefas e informações claras para passagem de telas, é eficiente para as funções propostas^(11)^.

Um produto ou serviço eficaz é aquele que consegue cumprir com os objetivos propostos, atende as necessidades reais dos usuários. Com relação a aplicativos móveis, é de extrema importância que o produto final consiga atingir as atividades propostas, levando em consideração se as funções foram suficientes. Ao realizar um teste de usabilidade, deve analisar a eficácia levando em consideração cada item apresentado, observando a presença de funções que não supriu as necessidades dos usuários^(12)^.

Foram avaliados 66 aplicativos disponíveis para pessoas com diabetes, utilizando a escala de *likert* de 5 pontos. Como resultado obteve-se a média de respostas entre 3 e 4, indicando que os aplicativos avaliados tiveram uma usabilidade moderada a boa, principalmente os que tinham grande facilidade de uso e linguagem clara^(13)^.

Como limitação do estudo, é importante mencionar que o Avazum ainda não está disponível para sistema iOS, sendo assim, não foi possível realizar o teste de usabilidade em pacientes que utilizam esse sistema para celular. Os próximos passos serão a expansão para o sistema iOS, tradução e adaptação para outros idiomas, como também a realização de novos testes para as atualizações feitas.

## CONCLUSÃO

Dessa forma, foi possível observar que os usuários demonstraram compreender todas as tarefas e conseguiram descrever o zumbido utilizando o aplicativo, evidenciando assim, que o Avazum mostrou efetivo para os objetivos propostos. Como também, as funções do aplicativo estão bem integradas, dispostas com clareza e com confortabilidade de uso, mostrando-se eficiente ao realizar as funções do app, além de bom índice de satisfação dos usuários ao utilizar o Avazum. Sendo um aplicativo promissor para auxiliar na avaliação do zumbido, encaminhamentos dos usuários e promover saúde através das orientações.

## Anexo A. Questionário Usabilidade

Satisfação

1.1 System Usability Scale-SUS (BROOKE, 1996)

Sobre sua satisfação em relação ao aplicativo, responda:

1 Discordo totalmente

2 Discordo

3 Neutro

4 Concordo

5. Concordo totalmente

1. Eu gostaria de usar este aplicativo com frequência

2. Achei o aplicativo desnecessariamente complicado

3. Achei o aplicativo fácil de usar

4. Achei que precisaria de apoio de uma pessoa mais especializada para ser capaz de usar este aplicativo

5. Achei que as diversas funções do aplicativo estão bem integradas

6. Achei que havia muita inconsistência neste aplicativo

7. imagino que a maioria das pessoas iria aprender a usar este aplicativo muito rapidamente

8. Eu me senti muito confiante usando o aplicativo

9. Eu precisaria aprender uma série de coisas antes que eu pudesse utilizar este aplicativo

1.2 Net Promoter Score

O quanto você recomendaria este aplicativo para alguém?

() 0 ()1 ()2 ()3 ()4 ()5 () 6 ()7 ()8 ()9 ()10

Eficácia de uso

2.1 Após o uso do aplicativo, você compreendeu as tarefas e orientações apresentadas pelo aplicativo? A linguagem é clara?

[ ] Sim, compreendi todas

[ ] Sim, compreendi apenas algumas

[ ] Não compreendi

No caso de resposta para as duas últimas alternativas, defina o grau de severidade do problema:

0 - sem importância

1 - Problema cosmético/aparência

2 - Problema simples

3 - Problema grave

4 - Problema Catastrófico

Qual sugestão de correção?

2.2 Você conseguiu descrever o zumbido que sente respondendo às questões apresentadas no aplicativo?

[ ] Sim

[ ] Em parte

[ ] Não

No caso de resposta para as duas últimas alternativas, defina o grau de severidade do problema:

0 - sem importância

1 - Problema cosmético/aparência

2 - Problema simples

3 - Problema grave

4 - Problema Catastrófico

Qual sugestão de correção?

2.3 As orientações no aplicativo sobre zumbido foram relevantes/interessantes para sua realidade?

[ ] Sim

[ ] Em parte

[ ] Não

No caso de resposta para as duas últimas alternativas, defina o grau de severidade do problema:

0 - sem importância

1 - Problema cosmético/aparência

2 - Problema simples

3 - Problema grave

4 - Problema Catastrófico

Qual sugestão de correção?

2.4 O encaminhamento para seguimento do atendimento com profissionais foi direcionado e de fácil acesso

[ ] Sim

[ ] Em parte

[ ] Não

No caso de resposta para as duas últimas alternativas, defina o grau de severidade do problema:

0 - sem importância

1 - Problema cosmético/aparência

2 - Problema simples

3 - Problema grave

4 - Problema Catastrófico

Qual sugestão de correção?

Eficiência de uso

3.1 Visibilidade dos elementos do aplicativo

A organização visual das imagens está adequada

[ ] Sim

[ ] Em parte

[ ] Não

No caso de resposta para as duas últimas alternativas, defina o grau de severidade do problema:

0 - sem importância

1 - Problema cosmético/aparência

2 - Problema simples

3 - Problema grave

4 - Problema Catastrófico

Qual sugestão de correção?

O tamanho das fontes utilizadas são legíveis são adequadas e legíveis

[ ] Sim

[ ] Em parte

[ ] Não

No caso de resposta para as duas últimas alternativas, defina o grau de severidade do problema:

0 - sem importância

1 - Problema cosmético/aparência

2 - Problema simples

3 - Problema grave

4 - Problema Catastrófico

Qual sugestão de correção?

3.2 Utilização da linguagem

Os enunciados são suficientes para realização das tarefas propostas no aplicativo

[ ] Sim

[ ] Em parte

[ ] Não

No caso de resposta para as duas últimas alternativas, defina o grau de severidade do problema:

0 - sem importância

1 - Problema cosmético/aparência

2 - Problema simples

3 - Problema grave

4 - Problema Catastrófico

Qual sugestão de correção?

As informações de uso são claras e possibilitam a passagem de fases no aplicativo

[ ] Sim

[ ] Em parte

[ ] Não

No caso de resposta para as duas últimas alternativas, defina o grau de severidade do problema:

0 - sem importância

1 - Problema cosmético/aparência

2 - Problema simples

3 - Problema grave

4 - Problema Catastrófico

Qual sugestão de correção?

3.4 Conforto

Você sentiu algum desconforto na utilização do aplicativo

[ ] Sim

[ ] Em parte

[ ] Não

No caso de resposta para as duas últimas alternativas, defina o grau de severidade do problema:

0 - sem importância

1 - Problema cosmético/aparência

2 - Problema simples

3 - Problema grave

4 - Problema Catastrófico

Qual sugestão de correção?
